# Rickets: An Overview and Future Directions, with Special Reference to Bangladesh: A Summary of the Rickets Convergence Group Meeting, Dhaka, 26–27 January 2006

**Published:** 2008-03

**Authors:** Thierry Craviari, John M. Pettifor, Tom D. Thacher, Craig Meisner, Josiane Arnaud, Philip R. Fischer, Rickets Convergence Group

**Affiliations:** 1 Centre Hospitalier de Gap, Gap, France; 2 University of Witwatersrand, Johannesburg, South Africa; 3 Jos University Teaching Hospital, Jos, Nigeria; 4 Cornell University, Ithaca, New York, USA; 5 Centre Hospitalier Universitaire de Grenoble, Grenoble, France; 6 Mayo Clinic, Rochester, 200 First Street SW, Minnesota 55905, USA

**Keywords:** Bones, Calcium, Calcium deficiency, Child, Epidemiology, Ossification, Rickets, Vitamin D, Vitamin D deficiency, Bangladesh

## Abstract

Rickets has emerged as a public-health problem in Bangladesh during the past two decades, with up to 8% of children clinically affected in some areas. Insufficiency of dietary calcium is thought to be the underlying cause, and treatment with calcium (350–1,000 mg elemental calcium daily) is curative. Despite this apparently simple treatment, little is known about the most appropriate management of bone deformities of affected children, and further studies are needed to determine the details of dosing and duration of calcium therapy, the role of bracing, and specific indications for surgical intervention. Effective preventive measures that can feasibly reach entire communities are needed, and these may differ between various affected regions.

## INTRODUCTION

Rickets is a condition associated with bone-deformity due to inadequate mineralization in growing bones (1,2). While some cases relate to hereditary syndromes, renal disease, or use of medication, rickets in the world mostly stems from nutritional insufficiency (3). Nutritional rickets is prevalent throughout much of the developing world and is again being increasingly seen in more affluent countries (3). Rickets has become common in some parts of Bangladesh during the past two decades. Indeed, the lay press has lamented the pain, deformity, and disability due to rickets (4), and one recent report even claimed that there were 5,000,000 affected children in Bangladesh (5).

In an effort to review current knowledge about nutritional rickets and to prioritize ongoing research, 135 people gathered in Dhaka in January 2006 for an International Congress on Rickets. Fourteen clinician-scientists—as part of the Rickets Convergence Group—provided plenary presentations, and other delegates contributed to lively discussions. Arising from material presented at this congress, this article provides an overview of the history, epidemiology, clinical findings, treatment, and prevention of nutritional rickets from both global and Bangladeshi perspectives. In so doing, an agendum for future research is proposed.

### History and epidemiology of rickets—globally and in Bangladesh context

Rickets was first reported in the mid-1600s in Europe (6). Glisson and others described typical findings of bone-deformity with curving of the legs. Rickets continued to be reported during successive centuries. By the 1800s, sunlight (ultraviolet radiation) and cod-liver oil were found to be effective in treating rickets, and in the early 1900s, vitamin D was isolated and found to be the essential ingredient of this oil (7).

With introduction of the supplementation of vitamin D, rickets became rare in industrialized nations during the 20^th^ century (8). At the end of the last century, however, two striking things happened. First, nutritional rickets ‘re-emerged’ as an important and widely-seen problem in North America (9,10). Second, rickets was prevalent in economically-disadvantaged parts of the world where vitamin D deficiency was not commonly found (3).

### Who does get rickets now?

In North America, rickets is most commonly seen in children with relatively more pigmented skin, who are exclusively breastfed (3). In Australia and Europe, rickets is mostly identified in immigrant populations from the Middle East and the Indian subcontinent (3). Most affected patients have symptoms within the first 6–12 months of life, and there is not a specific gender bias. In the Middle East, rickets is often seen in sun-protected children of vitamin D-deficient mothers, but it can present as bone-problems in later years of childhood (3). In sun-exposed regions of Asia and Africa, rickets typically presents during the second or the third year of life (3).

### Causes of rickets

Looking back even to the 1600s, nutritional rickets has traditionally been attributed to vitamin D deficiency relating to reduced exposure to sunlight, resulting from crowded living conditions under skies polluted by the detrimental products of industrialization (11). It has, however, also been suggested that the true cause of rickets in the 1600s might have been wet-nursing (use of mother's substitutes for nursing infants) by women with calcium-poor breastmilk (12). In North America and Europe, rickets was essentially eradicated during the mid-1900s with either vitamin D fortification of beverages, or cod-liver or administration of vitamin D oil solution—again supporting the notion that vitamin D was the cause. Even into the 1990s, calcium insufficiency was not accepted as a plausible cause of rickets in human beings (13). Nonetheless, there had been reports of calcium deficiency associated with rickets in South Africa (14) and Nigeria (15). By the late 1990s, evidence accumulated that low intake of dietary calcium was, indeed, important in the pathogenesis of rickets (16-18). In fact, calcium insufficiency was even suspected to contribute to some apparent vitamin D deficiency-related rickets seen in North America (19).

### Prevalence of rickets around the world

Rickets has been reported in dozens of countries during the past three decades (3). In some places, nutritional rickets is merely reported sporadically (3), while in other areas, up to 9% of the childhood population is clinically affected (20,21).

*History and epidemiology of nutritional rickets in Bangladesh***:** Rickets was first brought to broad attention in 1991 by workers from Social Assistance and Rehabilitation of the Physically Vulnerable visiting the Chakaria region of southeastern Bangladesh after a devastating cyclone. An informal village survey found that approximately 1% of children had rachitic deformities. Focus groups and local informants suggested that rickets was ‘new’ and had not been seen before the early 1970s. Children in Chakaria received care at the Memorial Christian Hospital, and 441 children with rickets were registered during 1991–1997. Anecdotally, outcomes of medical treatment with vitamin D were disappointing, and deformities recurred in children subjected to orthopaedic surgery. In 1994, French physicians, with Les Amis des Enfants du Monde, evaluated patients in communities from Chittagong to Moheskhali and identified rickets in 4.5% of them. Typically, findings of rickets were reported as beginning in the second and the third year of life. In 1997, academicians from Cornell University and other American institutions were apprised of the situation. A collaborative assessment revealed that rickets was more common than suspected in Chakaria; it was not generally associated with vitamin D deficiency but was related to insufficiency of dietary calcium (22). An international ‘Rickets Consortium’ was formed to stimulate collaborative research and practical interventions (23). This group subsequently reformed as the current Rickets Convergence Group which serves as a repository of information and a source of expertise to facilitate ongoing work relating to rickets in Bangladesh.

The Helen Keller International (Dhaka, Bangladesh) conducted a nationwide survey in 2000 and repeated it in 2004. Rickets was identified as visible varus and/or valgus deformities in children aged 1–15 year(s). Nationally, rachitic deformities were found in 0.26% of 21,571 surveyed children in 2000 and in 0.12% of 10,005 surveyed children in 2004. Rickets was found in more than half of the subdistricts with the highest prevalence being found in Sylhet (North-East) and Chittagong (South-East) divisions. The highest prevalence (1.4%) among 1 to 15 year(s) old children with visible rachitic deformities was found in the Cox's Bazaar subdistrict. A survey of all inhabitants in Chittagong carried out by the Bangladesh Rural Advancement Committee found rachitic deformities in 0.9% (24). A more detailed survey conducted by the Institute of Child and Mother Health in the Chittagong division found that 8.7% of children had at least one clinical finding indicative of rickets; 4% had deformities of the lower limb suggestive of rickets; 0.9% had radiological evidence of active rickets; and 2.2% had elevated levels of serum alkaline phosphatase (21). Rickets was not, however, identified in the indigenous populations in the Hill Tracts.

In Bangladesh, results of initial studies suggested that vitamin D deficiency was not a major causal factor in the prevalent rickets, and calcium deficiency is assumed to be the primary aetiologic factor (22). In Bangladesh, an emphasis on increasing the production of rice was associated with decreased rotation and variation of agricultural products and with decreased dairy production. While overall protein-energy malnutrition has become less common, the diet is less varied than it was three decades ago, and the diet contains less calcium. Nonetheless, less than 10% of children—all of whom seem to be calcium-deprived—actually develop clinical rickets. Boys seem to be more likely to develop rachitic deformities than girls, and rickets is associated with larger family size and less maternal education. Rickets is associated with respiratory illness but not with malaria or anaemia. Similarly, toxins, food patterns, and overall nutritional status are not associated with the prevalence of rickets among Bangladeshi children (25). The relationship between rickets and diarrhoea remains controversial. Genetic factors that potentially impact the risk of nutritional rickets have not been studied.

### Clinical features of rickets in Bangladesh and beyond

The clinical features of rickets are similar around the world, but the age of presentation and the risk of hypocalcaemic symptoms, such as tetany, vary depending on the age of presentation and the relative importance of vitamin D (versus calcium) deficiency in different populations. In areas where vitamin D deficiency is more common, rickets usually presents in the first year of life often with clinically-significant hypocalcaemia. In parts of Africa and in Bangladesh (where calcium deficiency accounts for much of the prevalent nutritional rickets), rickets usually presents from the second year of life, and hypocalcaemic tetany is much less commonly seen.

**Fig. 1:**
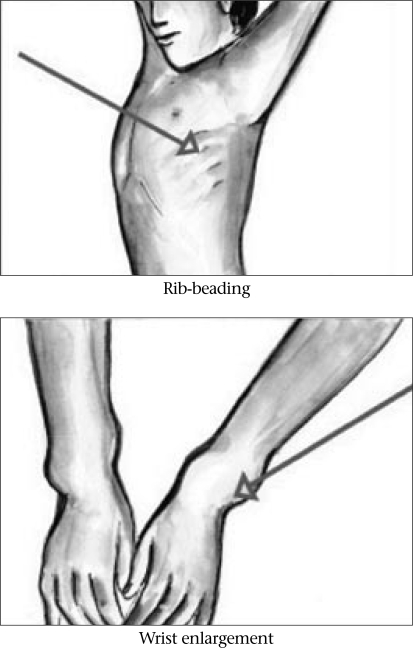
Clinical features of rickets

**Fig. 2:**
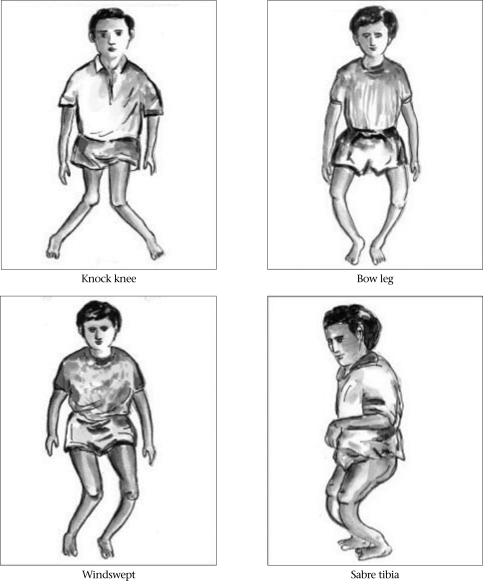
Clinical features of rickets: leg-deformities

Growth plates become soft as a result of diminished mineralization. With weight-bearing, gravitational pressure causes soft bones to curve in response to forces exerted across joints. Thus, the long bones of the leg curve—becoming ‘bow legs’ or, show up later onset of rickets in the form of ‘knocked knees’ (26). Metaphyses expand laterally such that wrists and ankles can be palpably widened. Costochondral junctions also expand with demineralized bone structures, and beading or pearling of the chest-wall is noted. Fontanels close late, and teeth erupt later than in other children (Fig. 1 and 2).

The use of specific physical examination criteria for a diagnosis of rickets, is, however, difficult. Determination of ‘wide’ wrists and ‘beaded’ ribs is a subjective experience in subtle cases. There are broad ranges of ‘normal’ lower limb curvature, fontanel closure, and tooth eruption. A Nigerian study has, however, given some basis for a clinical diagnosis of rickets when alkaline phosphatase levels and wrist/knee radiographs are not available (26). Specifically, for children with deformities of the lower limb, the finding of at least three of five features (age less than 5 years, short stature, leg pain with walking, wide wrists, costal beading) identified 87% of children with active rickets.

When resources are available, laboratory and radiologic testing should be used for confirming the diagnosis and aetiology of rickets. The serum alkaline phosphatase level is elevated when rickets is active, and x-rays of the knees and wrists show widened epiphyses with cupping and fraying of the metaphyseal border. Serum parathyroid hormone concentrations are usually elevated. With vitamin D-deficiency rickets, the 25-hydroxyvitamin D level is low, typically below 10 ng/mL (25 mmol/L). Without vitamin D deficiency, calcium deficiency stimulates elevation of the 1,25-dihydroxyvitamin D level, while the 25-hydroxyvitamin D levels remain normal or near-normal.

Rickets can be devastating to children. Affected children potentially experience delays in learning to walk, pain and fractures, and crippling deformities. In addition, rickets dramatically increases the risk of pneumonia (27), a condition which accounts for significant amounts of childhood mortality in developing regions of the world.

### Treatment of rickets around the world and in Bangladesh

Without medical management, rachitic leg-deformities can persist and worsen. Effective management of rachitic children begins with an appropriate diagnosis. When rickets is biologically active (elevated serum alkaline phosphatase level, x-rays with widened, cupped, frayed metaphyses), curative therapy is needed. This can consist either of supplementation of vitamin D (when the 25-hydroxyvitamin D level is known to be low or, when testing is not available, when the child is young with a history of limited sunlight exposure) or of replacement of calcium (when the 25-hydroxyvitamin D level is normal or, when testing is not available, when the child presents at an older age without a history of limited sunlight exposure) (28).

Doses of vitamin D for treatment are much higher than the doses needed to prevent rickets. When rickets is due to vitamin D deficiency, treatment may be initiated with vitamin D (5,000 units per day for several months).

For calcium-deficiency rickets, various doses have been used. In a Nigerian study, supplement of 1,000 mg of elemental calcium daily (noting that the elemental calcium makes up only a small part of some calcium salts) was effective when used for six months (17). In another study in Nigeria, 350 mg of elemental calcium daily was effective (29). In India, 1,000 mg of calcium as a daily treatment was adequate (30). Responses to treatment must be followed, and ongoing adequacy of calcium and vitamin D intake must be ensured.

**Fig. 3:**
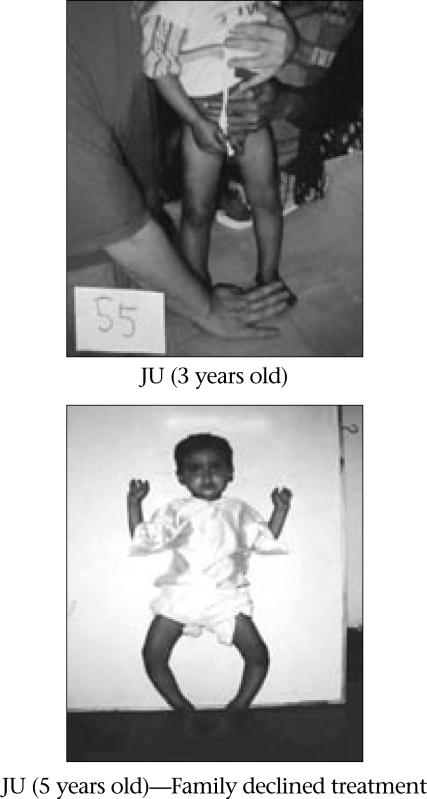
Evolution of rickets deformities without treatment

**Fig. 4:**
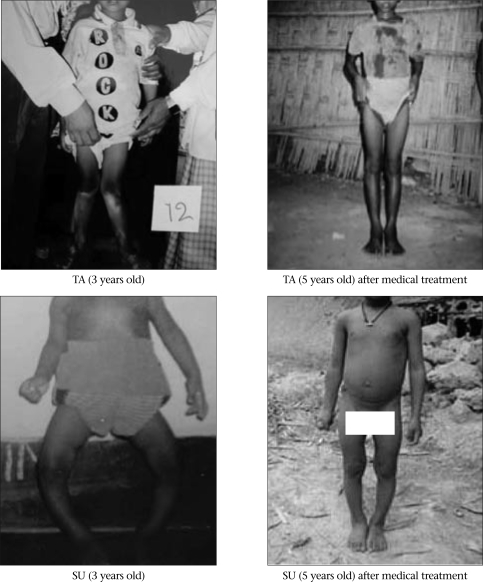
Response of rachitic deformities with medical treatment

Once medical treatment has prompted correction of biologically-active rickets (alkaline phosphatase levels and x-rays normal, or at least six months later when confirmatory testing is not available), the focus of treatment shifts to providing for the restoration of deformed extremities to functional alignment. With ongoing mineralization and weight-bearing, even severe deformities can improve (Fig. 3 compared to Fig. 4). Nonetheless, widening of wrists and beading of ribs can persist even after adequate medical treatment. Considerable ligamentous laxity may accompany marked leg-deformities. Associated with medical treatment, some clinical investigators suggest that braces be applied to support the limbs and to encourage straighter longitudinal growth. No comparative studies have been reported to confirm the usefulness (or not) of bracing.

When severe deformities persist despite medical therapy and ongoing longitudinal growth, surgical therapy can be considered. Evidence-based guidelines are not, however, available to guide the selection of children for surgery or to determine the timing of surgical intervention.

Ideally, rickets should be addressed by a community intervention, impacting all areas of life. To this end, the Chakaria Disabled Centre seeks to provide community education about the prevention and treatment of rickets. Through international collaborations, public-health education is provided. Consultation and diagnostic evaluation are provided for children, and therapeutic regimens are initiated. Even in traditional health centres, the management of rickets should fit within the framework of the Integrated Management of Childhood Illness programme (31) in such a way that children get comprehensive medical help.

How should additional calcium be delivered to rachitic children in Bangladesh? It is now known that increased intake of calcium provides curative treatment for many children. Would dietary calcium or non-pharmaceutic supplements be adequate? Studies are underway to look at the value of nutritional advice to improve intakes of dietary calcium to stimulate a curative response. Limestone (readily available in Bangladesh as ‘*choon*’—calcium hydroxide when melted in water) could be feasible, acceptable product to provide therapeutic doses of calcium. Increasing the intake of sesame seeds, green-leafy vegetables, crushed fish (containing the bones), and dairy products (if affordable and desirable) might also provide adequate increases in calcium intake to prevent rickets. Preliminary observational studies in Chakaria suggest that 90% of rachitic children with mild deformities of the leg (deformity angle less than 20^o^) improve during one year of nutritional education. In another retrospective review of 193 children, 75% had resolution of their rickets and reduction of the deformity with combined treatment, including nutritional advice and supplementation of 1,000 mg of elemental calcium per day, 17% stabilized, and 8% worsened despite the prescription of treatment (unpublished data). Compliance with six or more months of treatment was difficult, and it is unclear how many of the 8% of deteriorating children actually received the requested treatment. When there was success with treatment, it is not known how much of this improvement was due to the nutritional advice and how much was due to the other, incompletely controlled factors. Treatment was noted to be most successful in correcting deformity of the lower limb when it was instituted prior to six years of age.

**Fig. 5:**
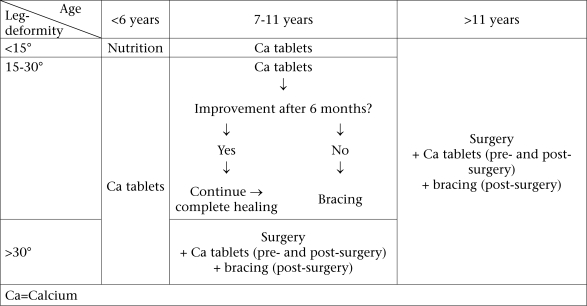
Graded treatment proposal for children with calcium-deficiency rickets

With medical management of rickets in order, workers at the Chakaria Disabled Centre are testing therapeutic regimens that could guide decisions about the timing of brace and surgical treatment. We recommend that rachitic children, aged less than six years, with less than a 15-degree angular deformity, should receive nutritional advice for six months. If worsening, they should receive supplementation of calcium. Rachitic children, aged less than six years, with more than a 15-degree angular deformity, should receive medication, such as calcium, and have at least one year of follow-up before suggesting bracing or surgery (Fig. 5). Children, aged 7–11 years, with angular deformities of 15–30 degrees could be considered candidates for bracing if treatment with calcium fails. Retrospectively, bracing was associated with improvement in 60% of children treated in this manner (unpublished data). Children, aged over six years, with an angular deformity of greater than 30 degrees, and children, aged over 11 years, with angular deformities greater than 15 degrees, could be considered surgical candidates. Surgeons can choose or combine osteotomy and epiphyseal scrapping or stapling as corrective measures depending on the age of the child and the degree of deformity. Anecdotally, bracing is helpful post-operatively to prevent recurrent deformity. Rates of infection are very low with surgery (<5%) with better results being seen with increasing surgeon's experience and when operations were done during the dry season. Over 1–4 year(s) of follow-up, 92% of 65 operated patients in Bangladesh had favourable (resulting angular deformity of 15 degrees or less) outcomes. Prospective evaluation of this graded selection of surgery is underway.

### Prevention of rickets

Compared to treatment of affected children, prevention of rickets is clearly better for children, desirable for communities, and possibly less expensive for society.

It is essential to identify the appropriate target population and their nutritional need before preventive interventions against rickets. In the United States, based on the known epidemiology of the resurgence in (at least some cases, vitamin D-deficient) rickets, the appropriate target would currently be to increase supply of vitamin D to exclusively-breastfed infants with darkly-pigmented skin and to their mothers during pregnancy. In Nigeria, the appropriate target would be infants and young children who suffer from calcium insufficiency. In India, there is evidence that young children need more calcium, while pubertal girls are most at risk of vitamin D-deficiency rickets (perhaps due to cultural habits limiting exposure to sunshine) (30). In Bangladesh, it seems that the appropriate target population would be older infants and young children who get adequate exposure to sun but need more calcium.

The appropriate ‘dose’ of preventive product must be identified. For vitamin D, children should receive the equivalent of 200–400 IU per day to prevent rickets. Alternatively, in temperate climates, exposure of the face and head to approximately 60 minutes of sunshine per week is probably adequate, and less exposure would be needed in areas nearer to the equator. Recommended intakes of calcium vary by age: in North America 210 up to six months of age, 270 from 7 to 12 months of age, 500 from one to three years of age, 800 from four to eight years of age, and 1,300 during the pubertal years (32). It must be remembered that the actual amount of elemental calcium varies with the type of calcium salt being used (1,000 mg of elemental calcium corresponds to 15,500 mg of calcium glubionate, 11,000 mg of calcium gluconate, 7,700 mg of calcium lactate, 4,700 mg of calcium citrate, and 2,500 mg of calcium carbonate) and that the bioavailability of calcium also depends on the type of calcium salt. Calcium carbonate can be taken as pills or as powdered limestone, with the limestone being only about 0.04% as expensive in many areas.

With the population appropriately selected and with the required intervention identified (usually either vitamin D or calcium), preventive strategies can be planned. In the middle decades of the last century, vitamin D deficiency was essentially eradicated by adding vitamin D to commercially-provided infant formulae and dairy products. Similarly, iodine deficiency was effectively eradicated by adding iodine to commercially-produced salt. In China, education about rickets was effective in reducing the prevalence of rickets, although it is not clear if the advised recommendations were actually implemented (33). In Nigeria, preliminary results suggest that rickets was less common when children aged 12–24 months were given either 400 mg of elemental calcium daily as tablets or the equivalent amount of calcium in dried ground fish added to porridge. Anecdotally, even small amounts of calcium included in daily porridge were found to be associated with a lower risk of rickets in an unpublished study in Bangladesh.

In rural areas, most young children grow up on diets devoid of commercial infant products. It is, thus, challenging to find a ‘point source’ at which vitamin D or calcium can be introduced in a way that would reach all children at risk in a developing country. Therefore, it makes sense to try to provide community-wide (or even nationwide or regionwide) education to try to increase the habitual intake of calcium in areas where calcium is widely deficient in the diets of young children.

Interventions targeting food systems might impact entire communities. The food system in the Chakaria region of Bangladesh was evaluated in an effort to identify household-level risk factors for rickets (25). Interestingly, most children had calcium-deficient diets containing less than 50% of the recommended amount of calcium, but rickets was only seen in a subset of houses. Adding lime (calcium) to the acid soils would not greatly increase the amount of calcium in plants; the additional calcium would increase the growth and health of the plants increasing biomass but not substantively increasing actual calcium content of the individual edible plant-parts. Efforts are underway to see if community-based drama can be effectively used for shifting dietary choices of the household [to either eat more small fish with the bones still in, eat more calcium-rich foods, such as cowpea, or add ‘*choon*’ (locally-used limestone) as a ‘seasoning’ to infant porridges]. Preliminary results suggest that both drama and video-based educational programmes stimulate discussion and learning but that only 12–16% of participants actually changed their food preparation in the recommended fashions.

## SUMMARY

Nutritional rickets is still frequently seen in many parts of the world. While vitamin D deficiency causes rickets in areas where either latitude is associated with relatively decreased exposure to sunlight or cultural habits block exposure to sunlight, calcium deficiency has emerged as an important cause of rickets in parts of Africa and Asia, including Bangladesh. Calcium-deficiency rickets typically presents after the first year of life with deformities of the leg, widened wrists, and beaded ribs. Rickets carries a high risk of developing pneumonia. The diagnosis is suspected clinically and can be confirmed with elevated levels of serum alkaline phosphatase and/or radiographic evidence of widened, cupped, frayed epiphyses. When available, testing of vitamin D metabolites (25 hydroxy- and 1,25 di-hydroxy-vitamin D) can help distinguish between the possible causes of nutritional rickets. Treatment is effective by providing adequate amounts of the missing nutrient(s), and preventive programmes are needed. Further research is needed to: (a) determine the best way to accurately diagnose rickets and its cause in areas without complete laboratory and radiologic facilities, (b) determine the best dose and duration of treatment of calcium, (c) determine the appropriate indications and timing of surgical interventions, (d) identify the value of bracing for children with medically-treated rickets, (e) determine effective means of preventing rickets, and (f) test strategies by which preventive interventions can be widely implemented.

The Rickets Convergence Group continues to investigate rickets and to study preventive and therapeutic interventions. Another international conference on rickets is being planned for Dhaka, Bangladesh.
